# Probiotics Exert Colonization Resistance Against *F. nucleatum* subsp. *polymorphum*: Disruption by Antibiotics and Underlying Molecular Mechanisms

**DOI:** 10.3390/microorganisms14050965

**Published:** 2026-04-24

**Authors:** Wenling Huang, Jingheng Liang, Poukei Chan, Zhaohui Liu, Lihong Guo

**Affiliations:** 1 Hospital of Stomatology, Sun Yat-sen University, Guangzhou 510055, China; 2 Guangdong Provincial Key Laboratory of Stomatology, Sun Yat-sen University, Guangzhou 510055, China

**Keywords:** *Fusobacterium nucleatum*, colorectal cancer, probiotics, colonization resistance, antibiotics

## Abstract

*Fusobacterium nucleatum* (*F. nucleatum*), a key oral pathogen, promotes colorectal cancer (CRC) progression via gut translocation. Although gut probiotics provide colonization resistance against pathogens, antibiotic-induced dysbiosis may facilitate *F. nucleatum* integration and increase the risk of CRC. The mechanisms underlying probiotic—*F. nucleatum* antagonism and antibiotic modulation remain unclear. A 33-strain probiotic consortium and *F. nucleatum* subsp. *Polymorphum* (F. *polymorphum*) ATCC 10953 were co-cultured. The inhibitory effects of probiotics on *F. nucleatum* and the impacts of antibiotics (ABXs) on the microbial community structure in the co-culture system and on the probiotic-mediated inhibition of *F. nucleatum* were evaluated using spent medium assays, plate confrontation tests, growth curves, qRT-PCR, metagenomic sequencing, and transcriptomics. Hydrogen peroxide/pH/lysine assays and coaggregation models were performed to probe the associated mechanisms. Probiotics strongly inhibited the growth of *F. nucleatum* in a dose-dependent manner, primarily via organic acids, while *F. nucleatum* enriched amino acid/vitamin biosynthesis pathways without major growth suppression. Antibiotics weakened probiotic antagonism, shifted species abundance (↓ *L. plantarum*, ↑ *L. paracasei*), induced adaptive stress responses in *F. nucleatum* (↑ nucleotide metabolism, propanediol degradation, *pdxS*), and reduced lysine biosynthesis. Lysine supplementation restored probiotic abundance and disrupted *F. nucleatum* coaggregation. Multi-strain probiotics exert potent colonization resistance effects against *F. nucleatum*, mainly through organic acids and metabolic interference. Antibiotic-induced dysbiosis impairs this protective effect and may promote the persistence of *F. nucleatum*, which has been implicated in CRC risk. Targeted probiotic strategies may offer novel preventive approaches.

## 1. Introduction

Colorectal cancer (CRC) is now considered one of the most common malignant tumors. In 2022, the global incidence of CRC ranked third, while its mortality rate ranked second, posing a serious threat to human health [1].

*Fusobacterium nucleatum* (*F. nucleatum*), a Gram-negative obligate anaerobe, mainly colonizes the oral cavity in healthy populations. Due to its bridging bacteria role, it represents a key factor in the colonization and growth of periodontal pathogens [2], and is considered to be closely related to the occurrence and development of periodontitis.

In the past few years, studies have shown that *F. nucleatum* can localize to CRC tissues through the oral–intestinal pathway and periodontitis-related blood circulation, promoting the development of CRC [3,4]. Numerous studies have indicated that *F. nucleatum* is closely related to the development of CRC and treatment resistance [5,6]. Some studies have proposed a “double-hit” model for CRC occurrence, in which somatic mutations serve as the first hit, while *F. nucleatum* acts as the second hit, exacerbating tumor development through its enrichment in CRC tissues. Therefore, *F. nucleatum* is identified as a “promoter” of CRC [5,7].

Recent taxonomic studies have refined the subspecies classification of *F. nucleatum* and revealed distinct associations with CRC. Specifically, *F. nucleatum* subsp. *animalis*, subsp. *vincentii*, and subsp. *watanabei* are the predominant subspecies enriched in CRC tissues, whereas subsp. *polymorphum* is more commonly found in the oral cavity and is less frequently detected in CRC specimens [8]. Nevertheless, subsp. *polymorphum* remains a representative oral isolate [9] and has been widely employed to study the initial colonization, interspecies interactions, and metabolic adaptations of fusobacteria along the oral–gut axis [10,11].

It is noteworthy that the precise mechanisms by which *F. nucleatum* translocates from the oral cavity to the intestine, colonizes the intestinal microbiota, and subsequently causes disease remain unclear.

Gut microbiota is an important biological barrier in the human body, playing a key role in host immunity and nutrition. Dysbiosis of the gut microbiota is one of the main risk factors for CRC occurrence [12]. The gut microbiota can exhibit antagonistic properties against pathogens, known as colonization resistance. The ecological diversity of the gut microbiota and its interactions drive the community’s colonization resistance [13].

Studies have demonstrated that the intestinal probiotic community in healthy individuals shows significant resistance against *F. nucleatum*; meanwhile, in CRC patients, the intestinal microbial community shows significant imbalance, with reduced abundance of probiotic communities that maintain intestinal ecological stability and relative enrichment of *F. nucleatum*, thus promoting inflammatory responses and metabolic disorders [14].

Maintaining gut microbiota stability is crucial, and the widespread use of antibiotics is one of the most significant factors disrupting this balance. Large cohort studies have shown that antibiotic exposure is associated with increased CRC risk and exhibits dose dependence [15,16]. Studies have indicated that, after antibiotic use, the abundance of pathogens in the intestine, such as *Fusobacteria*, *Porphyromonas*, and *Enterobacteriaceae*, may increase; these bacteria are related to CRC and may form biofilms, promoting the initiation of carcinogenesis [17]. The estimated global antibiotic consumption in 2010 was 70 billion single doses, equivalent to 10 doses per person; this number continues to grow steadily, highlighting the severity of this public health problem [18].

Therefore, this study aimed to elucidate how the intestinal probiotic community interacts with *F. nucleatum* and whether microbiota dysbiosis caused by antibiotic abuse may facilitate the integration of *F. nucleatum* into the intestinal microbiota, potentially contributing to CRC risk. The above questions remain unanswered in the existing literature.

This study provides new ideas for precision microbiome intervention strategies based on probiotic communities (e.g., specific strain combinations and metabolite-targeted delivery), focusing on CRC prevention and treatment, as well as strategies for rebuilding the microbiota structure and function after antibiotic treatment.

## 2. Materials and Methods

### 2.1. Bacterial Strains and Culture Conditions

*F. nucleatum* subsp. *polymorphum* (*F. polymorphum*) ATCC 10953, *Lactiplantibacillus plantarum* ATCC 8014, and *Lacticaseibacillus paracasei* ATCC 334 were acquired from Guangdong Microbial Culture Collection Center (GDMCC, Guangzhou, China). Intestinal probiotics were acquired from Ranyi Technology Co., Ltd. (Shenzhen, China) (order number 1885748307538553773). The selected intestinal probiotics consisted of 33 well-characterized strains belonging to 9 genera (*Lactiplantibacillus*, *Lactobacillus*, *Lacticaseibacillus*, *Ligilactobacillus*, *Limosilactobacillus*, *Bifidobacterium*, *Lactococcus*, *Streptococcus*, *Pediococcus*). All genera and species included in this study possess Qualified Presumption of Safety (QPS) status according to the European Food Safety Authority (EFSA) [19], indicating that they are considered safe for deliberate use in food and feed. These taxa are also widely recognized as core probiotic genera by the International Scientific Association for Probiotics and Prebiotics (ISAPP) [20] and have been extensively documented in numerous human and animal studies regarding their ability to modulate the gut microbiota, inhibit pathogens, and confer health benefits [21,22].

The probiotics used in this study include *Lactiplantibacillus plantarum* LP4, *Lactiplantibacillus plantarum* CN2018, *Lactobacillus acidophilus* La28, *Lactobacillus acidophilus* L837, *Lacticaseibacillus paracasei* YMC1069, *Lacticaseibacillus paracasei* L578, *Lacticaseibacillus rhamnosus* RL519, *Lacticaseibacillus rhamnosus* LR863, *Lacticaseibacillus rhamnosus* L839, *Lacticaseibacillus casei* L560, *Swiss Lactobacillus* L551, *Lactobacillus gasseri* L838, *Ligilactobacillus salivarius* L663, *Limosilactobacillus fermentum* L665, *Lactobacillus bulgaricus* L574, *Lactobacillus bulgaricus* L8, *Limosilactobacillus reuteri* L840, *Bifidobacterium bifidum* TMC3115, *Bifidobacterium longum* subsp. *infantis* L998, *Bifidobacterium animalis* subsp. *lactis* BAL531, *Bifidobacterium longum* L693, *Bifidobacterium adolescentis* L996, *Bifidobacterium breve* L956, *Lactococcus lactis* S133, *Lactococcus lactis* S28, *Lactococcus lactis* S52, *Lactococcus lactis* S29, *Streptococcus thermophilus* S1, *Streptococcus thermophilus* S2, *Streptococcus thermophilus* S131, *Streptococcus thermophilus* S709, *Streptococcus thermophilus* S83, and *Pediococcus pentosaceus* S698.

The probiotics were anaerobically cultured in Brain Heart Infusion (BHI) medium (Difco, Detroit, MI, USA) at 37 °C (85% N_2_, 10% H_2_, 5% CO_2_). *F. nucleatum* was anaerobically cultured in BHI medium containing 5 g/L yeast extract (Thermo Fisher Scientific, Waltham, MA, USA), 0.4 g/L L-cysteine HCl (Amresco, Solon, OH, USA), 0.005 g/L hemin (Solarbio, Beijing, China), and 0.001 g/L vitamin K (Ronshyn, Shanghai, China) (BHIH medium) at 37 °C. *F. nucleatum* and the probiotics were cultured to the late logarithmic phase for later use.

### 2.2. Susceptibility of Probiotics and F. nucleatum to Antibiotics

The minimal inhibitory concentrations (MICs) of an antibiotic cocktail (ABXs, consisting of 1 g/L ampicillin, 1 g/L neomycin sulfate, 1 g/L metronidazole, and 0.5 g/L vancomycin) (Solarbio, China) [23], with respect to *F. nucleatum* or the probiotics, was determined via Clinical and Laboratory Standards Institute (CLSI)-recommended broth microdilution (M07) with minor modifications for anaerobes. The probiotic consortium was tested as a single standardized mixed inoculum (total 1.0 × 10^6^ CFU/mL), with the MIC defined as the lowest ABX concentration inhibiting visible growth of the entire pool. This pooled approach is consistent with recently validated P-AST methods for polymicrobial consortia that meet CLSI performance criteria [24,25].

### 2.3. Preparation of ABX-Pretreated Probiotics and Their Spent Medium

Probiotics were treated with ABXs at the MIC or half-MIC (hMIC) and cultured anaerobically at 37 °C for 24 h. Cells were harvested by centrifugation (4000× *g*, 15 min, 4 °C), washed three times with PBS, and resuspended in fresh BHI medium to a concentration of 10^8^ CFU/mL to obtain ABX-pretreated probiotic suspensions.

To prepare the spent medium of ABX-pretreated probiotics, the above bacterial suspensions were diluted 1:30 in BHI medium and incubated anaerobically at 37 °C for an additional 24 h. The cultures were then centrifuged (4000× *g*, 15 min, 4 °C) and the supernatant was filtered through a 0.22 μm sterile filter to obtain the cell-free supernatant (CFS). The CFS was mixed with BHIH medium at a volume ratio of 1:9 to prepare the final spent media, designated as SM_MIC and SM_hMIC. The spent medium from untreated probiotics was prepared following the same protocol and designated as SM.

### 2.4. Spent Medium Assay

A spent medium assay was used to examine the inhibitory effect of the probiotic (untreated or ABX-pretreated) or *F. nucleatum* CFS. The CFS was collected from late-logarithmic phase cultures via centrifugation (4000× *g*, 15 min, 4 °C) and 0.22 μm filtration.

For the probiotic CFS (from untreated or ABX-pretreated cells), serial dilutions in BHIH medium (1:9 to 1:1) were prepared. Then, 1 mL of *F. nucleatum* (10^9^ CFU/mL) was added to 10 mL of each dilution; *F. nucleatum* in BHIH medium alone served as the control.

For the *F. nucleatum* CFS, the same dilution protocol was applied using BHI medium, followed by the addition of probiotics, with probiotics in BHI medium serving as the control.

In a separate experiment, to test the effect of ABX pretreatment on spent medium activity, *F. nucleatum* (10^9^ CFU/mL, 1 mL) was added to 10 mL of SM, SM_MIC, or SM_hMIC (prepared as in Section 3) and cultured anaerobically for 24 h, with untreated *F. nucleatum* serving as the control.

In all setups, cultures were incubated anaerobically at 37 °C, and optical density at 600 nm was measured every 4 h using a microplate reader (Biotek, Winooski, VT, USA). Due to the prolonged (24 h) anaerobic incubation required for *F. nucleatum* and the probiotic consortium, and to minimize the contamination risk associated with repeated sampling from the same flask, we adopted an alternative design: for each time point, independent cultures (biological replicates, *n* = 3) were prepared and measured only once. This approach generates independent measurements, allowing for statistical analysis at each time point without the need to correct for repeated measures.

### 2.5. Plate Confrontation Test

**Figure 1:**
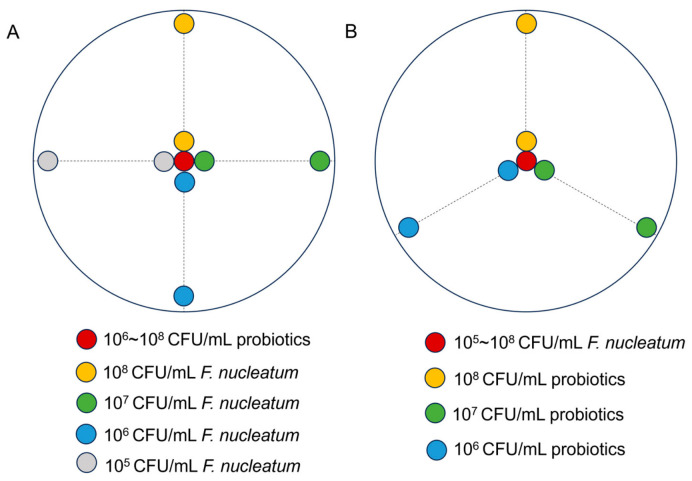
Diagram of the plate confrontation test. (**A**) Schematic illustration of the plate confrontation assay. The central spot represents the test strain (probiotics), the peripheral spot (40 mm away) represents the opposing test strain (*F. nucleatum*), and the negative control is the same as the peripheral strain but placed alone at the edge of the plate. The dashed lines indicate the approximate boundary of bacterial growth inhibition. (**B**) Reverse setup. The central spot represents *F. nucleatum*, the peripheral spot represents probiotics, and the negative control is placed at the edge of the plate as in (**A**).

The mutual inhibitory effects between probiotics and *F. nucleatum* were determined using the plate confrontation test. Probiotics were first treated with or without ABX, as described in Section 2.3. After treatment, the bacterial suspensions were adjusted to different concentrations (10^6^–10^8^ CFU/mL), and 10 μL of each was dropped onto the center of BHI blood agar plates. Then, 10 μL of *F. nucleatum* at different concentrations (10^5^–10^8^ CFU/mL) was dropped onto the plate placed 40 mm away from the center. *F. nucleatum* at the same concentration was dropped onto the edge of the plate as the negative control (Figure 1A).

Conversely, in the reverse setup, *F. nucleatum* (10^5^–10^8^ CFU/mL) was spotted at the center, with probiotic droplets placed 40 mm away, and a corresponding negative control was included (Figure 1B).

### 2.6. RNA Extraction and qRT-PCR from Co-Culture

Co-culture concentrations were selected based on preceding assays to ensure clear inhibition while retaining sufficient bacterial yield. Accordingly, *F. nucleatum* (10^6^ CFU/mL) was mixed with an equal volume of probiotics (10^8^ CFU/mL) and incubated anaerobically at 37 °C for 24 h, with a *F. nucleatum* monoculture serving as the control. Total RNA was extracted using RNAzol^®^ RT (MRC, Cincinnati, OH, USA) according to the manufacturer’s instructions, including lysozyme treatment, phase separation, and isopropanol precipitation. RNA was quantified using a NanoDrop 2000 spectrophotometer (NanoDrop Technologies, Wilmington, DE, USA). Reverse transcription was performed with PrimeScript™ RT Master Mix (TAKARA, Kusatsu, Shiga, Japan), followed by quantitative PCR using SYBR^®^ Premix Ex Taq™ II (TAKARA) on a LightCycler 96 system (Roche, Basel, Switzerland) to obtain cDNA. The 2^−ΔΔCt^ method was used to quantify fold changes relative to the *F. nucleatum* 16S rRNA gene. The 16S rRNA transcript level reflects the metabolic activity of *F. nucleatum*, as rRNA content is positively correlated with bacterial growth rate and ribosome synthesis activity [26,27,28]. Under standardized culture conditions, 16S rRNA-targeted RT-qPCR has been shown to correlate well with CFU counts [29,30]; therefore, in our controlled in vitro system, the RT-qPCR signal is interpreted as representing both physiological activity and, to a meaningful extent, relative bacterial abundance. The primer sequences were F-CTTAGGAATGAGACAGAGATG and R-TGATGGTAACATACGAAAGG.

The 16S rRNA transcript level reflects the metabolic activity of *F. nucleatum*, as rRNA content is positively correlated with bacterial growth rate and ribosome synthesis activity.

### 2.7. Metagenomic Sequencing and Analysis

Total DNA was extracted from co-culture and control samples using the TIANamp Bacteria DNA Kit (TIANGEN, Beijing, China), according to the manufacturer’s instructions. and then subjected to draft-genome sequencing on DNBSEQ-G400. Following the removal of low-quality sequences and adapters, the reads were de novo assembled using the MEGAHIT software v0.1-beta [31]. Assembled contigs with a length less than 300 bp were discarded in the following analysis. Genes were predicted over contigs using MetaGeneMark v3.38 [32]. Redundant genes were removed using CD-HIT v4.8.1 [33] based on identity and coverage cutoffs of 95% and 90%, respectively. To construct a gene abundance matrix, the Salmon v1.6.0 [34] software was used for quantification. Differentially enriched KEGG pathways were identified according to reporter scores. Alpha diversity was quantified by means of the Shannon index, chao1 index, and Simpson index using the relative abundance profiles at the gene, genus, and KO levels with the R package, respectively. Beta diversity was calculated using the Bray–Curtis distance based on the species-level relative abundance matrix. Principal coordinate analysis (PCoA) was performed for visualization. Raw data were deposited in the Sequence Read Archive (SRA) under Bioproject accession numbers PRJNA1415463 and PRJNA1415878.

Detailed metagenomic analysis parameters: Sequencing depth averaged 10 GB per sample. Three biological replicates were used per group (IP, Fnp-IP, Fnp-IP_hMIC, Fnp-IP_MIC). Assembly was performed using MEGAHIT v1.2.9. Gene prediction was conducted using MetaGeneMark v3.38. Redundant genes were removed using CD-HIT v4.8.1 with identity and coverage thresholds of 95% and 90%, respectively. Gene abundance was normalized using FPKM (Fragments per Kilobase per Million). For taxonomic classification, Kraken2 v2.1.2 was used with a custom database comprising NCBI NT (k2_pluspf_16gb_20240112) and UHGG v2.0; species abundance was estimated using Bracken2 v2.6.2. Alpha diversity and beta diversity were calculated using a species-level relative abundance matrix. For KEGG pathway enrichment, reporter scores were calculated according to the method of Patil and Nielsen [35]. Briefly, KO-level differential abundances were integrated into pathway-level Z-scores, which were then corrected by permutation (1000 times) to obtain a standard normal distribution; a reporter score with |Z| > 1.65 was considered significant (*p* < 0.05, one-tailed).

### 2.8. RNA Sequencing Transcriptome

RNA sequencing was conducted using the DNBSEQ platform (PE150 read length, average sequencing depth 4.36 Gb per sample). SOAPnuke (v1.6.5) was applied to filter reads and obtain clean reads; reads with adapters, more than 1% unknown bases, and low quality were removed. The clean reads were mapped to the Fusobacterium polymorphum ATCC 10953 reference genome GCF_037900625.1_ASM3790062v1 using HISAT2 V2.0.4. Gene expression levels were calculated using Bowtie2 V2.4.5 and RSEM V1.3.1. The library preparation strategy is illustrated in Figure S1. Briefly, total RNA was subjected to rRNA depletion using DNA probes that hybridize to rRNA, followed by RNase H digestion and DNase I treatment. After fragmentation, random N6 primers were used for reverse transcription, followed by second-strand cDNA synthesis. The double-stranded cDNA was end-repaired, A-tailed, and ligated to adapters. After PCR amplification, the product was denatured and circularized to obtain a single-stranded circular DNA library. The libraries were stranded. RNA was extracted from *F. nucleatum* monocultures after 24 h treatment with the probiotic supernatant or control medium. No probiotics were present during RNA extraction; thus, all sequenced reads originated from *F. nucleatum*. Three biological replicates were used per condition (Fn, YSJ, YSJ_MIC, YSJ_hMIC). All 12 samples passed read-level filtering (removal of adapters, low-quality reads, and reads with >1% N bases); no sample was excluded based on total reads, mapping rate, or correlation. Stringent criteria, including Log_2_FC > 1 and a false discovery rate (FDR < 0.001), were applied to filter differentially expressed genes (DEGs).

Data mining and figure presentation, including KEGG classification and GO classification analysis, were performed using the BGI in-house customized data mining system, Dr. Tom (https://biosys.bgi.com/). RNA sequencing data supporting the findings of this study were deposited in the NCBI SRA under accession number PRJNA1415531. qRT-PCR was used to validate changes in mRNA expression of DEGs. Gene-specific primer sequences are listed in Table S1.

### 2.9. Hydrogen Peroxide Content Determination and Depletion Experiment

The CFS of the probiotics was collected, and the content of hydrogen peroxide (H_2_O_2_) was determined using the hydrogen peroxide content detection kit, according to the manufacturer’s recommendations (Solabrio, Beijing, China).

The H_2_O_2_ in the probiotic CFS was depleted by 10 μg/mL catalase (Solarbio, Beijing, China). We added 300 μL of 10^9^ CFU/mL *F. nucleatum* to 3 mL of the probiotic CFS with or without depletion of H_2_O_2_. We then prepared 0.5% BHIH_ H_2_O_2_ medium based on the H_2_O_2_ concentration in the CFS, and added catalase to ensure complete H_2_O_2_ depletion. 0.5% BHIH_ H_2_O_2_ medium with or without hydrogen peroxide depletion was used as the negative control group.

The bacterial suspensions were cultured at 37 °C anaerobically for 8 h, 12 h, and 24 h, and the optical densities were measured at a wavelength of 600 nm using a microplate reader (Biotek, Winooski, VT, USA).

### 2.10. Effect of pH of Probiotic CFS on F. nucleatum

The pH of the probiotic CFS was determined, and was then adjusted to 7.45 by adding K_2_HPO_4_. The CFS with or without pH adjustment was mixed with BHIH medium at a 1:9 ratio. We added 300 μL of 10^9^ CFU/mL *F. nucleatum* to 3 mL of the probiotic CFS with or without pH adjustment. BHIH medium (adjusted to pH 3.45 or unadjusted) was used as the negative control.

The bacterial suspensions were cultured at 37 °C anaerobically for 8 h, 12 h, and 24 h, and the optical densities were measured at a wavelength of 600 nm using a microplate reader (Biotek, Winooski, VT, USA).

### 2.11. Lysine Content Determination and Supplementation Experiment

The CFS of the probiotics treated with or without ABXs and the CFS of *F. nucleatum* cultured with or without probiotics were collected, and the content of lysine was determined by using a lysine content detection kit, according to the manufacturer’s recommendations (Aidisheng, Yancheng, China). Based on the lysine concentration in the probiotic suspension, L-lysine was used to supplement the lysine concentration of the ABX-treated probiotic suspension to 11 mg/mL. Based on the lysine concentration in the co-culture suspension of probiotics and *F. nucleatum*, the lysine concentration in the *F. nucleatum* and ABX-treated probiotic suspension was supplemented to 15 mg/mL. The bacterial suspensions were cultured at 37 °C anaerobically for 24 h. Then, the number of probiotics with or without ABX treatment was confirmed by plate counting, and the metabolic activity of *F. nucleatum* was validated via qRT-PCR.

### 2.12. Establishment of Coaggregation Model

Coaggregation buffer (CAB) (150 mM NaCl, 1 mM Tris, pH 8.0, 0.1 mM CaCl_2_, 0.1 mM MgCl_2_) was prepared as previously described [36]. The intergeneric coaggregation model of probiotics and *F. nucleatum* was established as previously described [37,38,39]. Briefly, probiotics and *F. nucleatum* in the late logarithmic growth phase were washed twice and resuspended in CAB, adjusted to a final concentration of 10^9^ CFU/mL. The probiotics and *F. nucleatum* were allowed to coaggregate by mixing equal suspensions of bacterial cells in a reaction tube, which was then vortexed for 20 s and left undisturbed anaerobically at room temperature for 10 min. After incubation, coaggregated probiotics and *F. nucleatum* were pelleted via low-speed (200× *g*) centrifugation for 1 min, while non-coaggregated bacteria were dispersed in the supernatant, which was collected carefully and measured for OD_600nm_ (OD_IP-Fn_).

The degree of coaggregation was evaluated using the coaggregation index (CI), calculated according to the following formula: $CI=\frac{{OD}_{IP}+{OD}_{Fn}-{OD}_{IP-Fn}}{{OD}_{IP}+{OD}_{Fn}}\times 100\%$ [40,41]. OD_IP_ and OD_Fn_ represent the optical density of the probiotics and *F. nucleatum* at 600 nm, respectively.

### 2.13. Statistical Analysis

Statistical analysis and graphing were performed using Graphpad Prism 9.5.0. *t*-tests were used for inter-group comparisons, with *p* < 0.05 considered to indicate significant differences. Wilcoxon/Kruskal and KEGG pathway enrichment analyses (report score method) were performed to mine differences in species composition and functional composition between metagenomic samples. One-way analysis of variance (one-way ANOVA) was used for multi-group comparisons; if significant (*p* < 0.05), Dunnett’s correction and Tukey’s HSD were used for multiple comparisons. Dunnett’s correction and Tukey’s HSD were used to adjust *p*-values to *Q*-values, with *Q*-value < 0.05 considered to indicate significant differences.

## 3. Results

### 3.1. F. nucleatum Significantly Enriched Metabolic Pathways in the Probiotic Consortium

**Figure 2:**
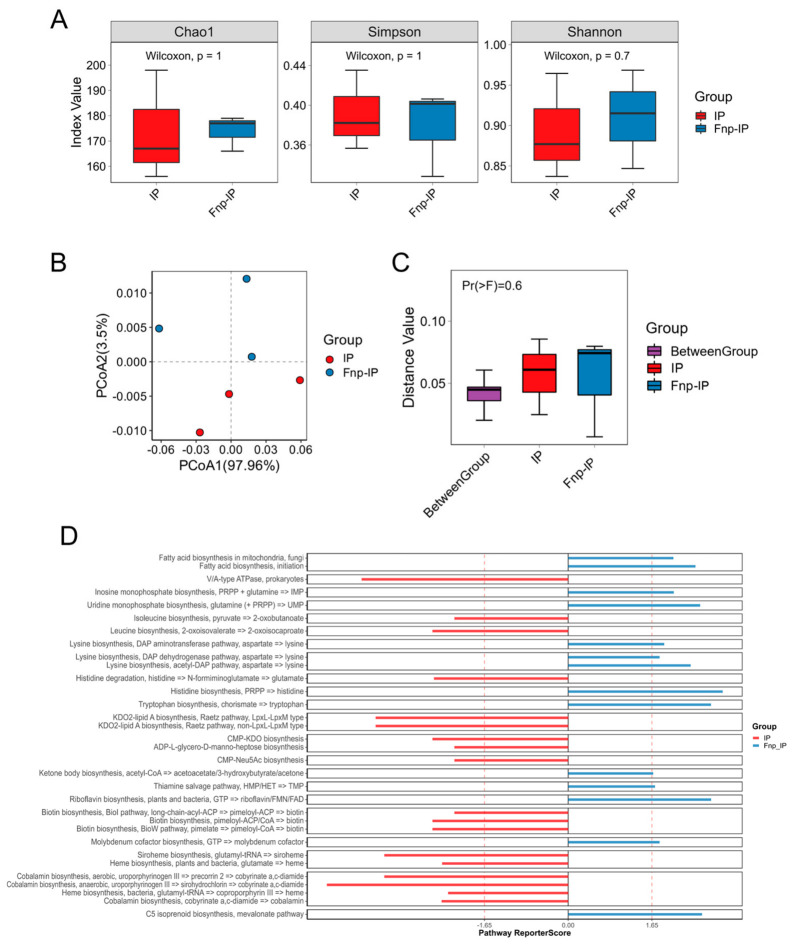
Metagenomic analysis of the probiotic consortium cultured with or without *F. nucleatum* (Fnp). (**A**) Alpha diversity (Shannon index) of probiotics alone (IP, *n* = 3) and probiotics co-cultured with Fnp (Fnp-IP, *n* = 3). Boxes show median ± IQR; whiskers indicate range. No significant difference was observed. (**B**) Principal coordinate analysis (PCoA) of beta diversity based on species abundance. Each dot represents one biological replicate (*n* = 3 per group). (**C**) PERMANOVA (Bray–Curtis, 999 permutations) detected no significant separation between IP and Fnp-IP (*p* = 0.6, R^2^ = 0.10). (**D**) KEGG pathway enrichment analysis. Significantly enriched pathways in Fnp-IP (blue) and IP (red) (*p* < 0.05). IP: probiotics alone; Fnp: *F. nucleatum*; Fnp-IP: co-culture of probiotics with *F. nucleatum*.

Metagenomic sequencing analysis was performed after co-culturing *F. nucleatum* with probiotics. Alpha diversity analysis showed (Figure 2A) no significant differences in species richness and evenness between the co-culture group (Fnp-IP) and the solo probiotic group (IP) (*p* > 0.05). Beta diversity was visualized via PCoA based on the Bray–Curtis distance. PERMANOVA (999 permutations) did not detect a statistically significant difference in overall community composition between the IP and Fnp-IP groups (*p* = 0.6, R^2^ = 0.10; Figure 2B,C).

KEGG pathway enrichment analysis showed (Figure 2D) that significantly enriched pathways in the Fnp-IP group included histidine biosynthesis, tryptophan biosynthesis, riboflavin biosynthesis, C5 isoprenoid biosynthesis, mevalonate pathway, fatty acid biosynthesis, and lysine biosynthesis, while enriched pathways in the IP group included isoleucine biosynthesis, biotin biosynthesis, cobalamin biosynthesis, siroheme biosynthesis, leucine biosynthesis, and histidine degradation.

### 3.2. Probiotics Significantly Inhibit the Growth of F. nucleatum, While F. nucleatum Does Not Obviously Affect Probiotic Growth

**Figure 3:**
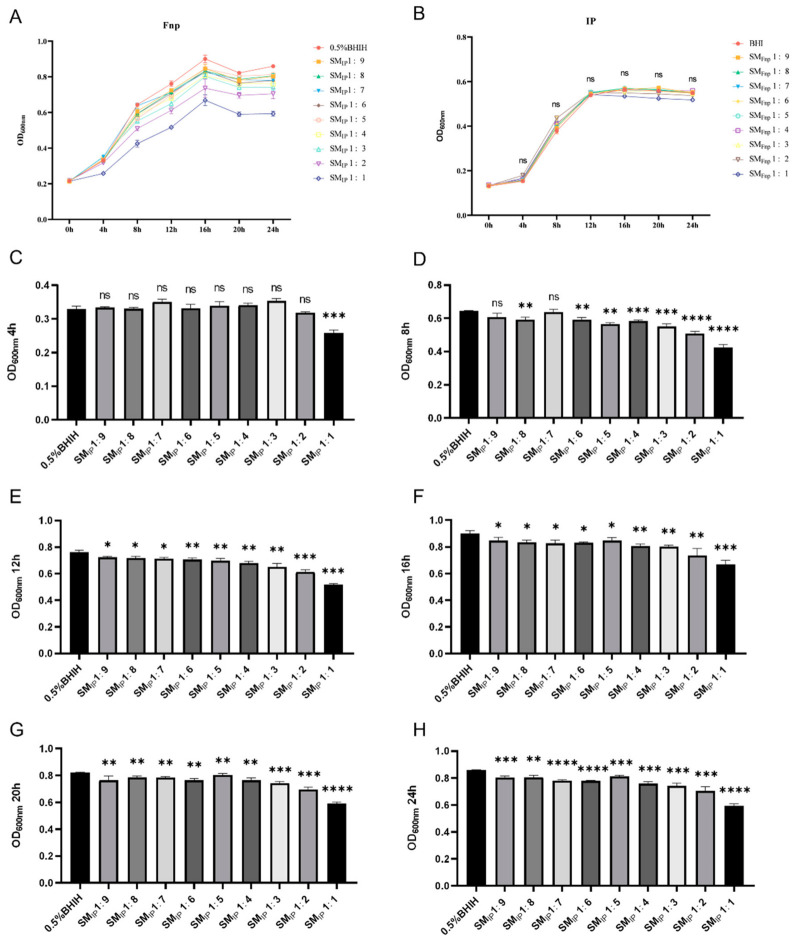
Growth of *F. nucleatum* and probiotics, and dose-dependent inhibition of *F. nucleatum* by probiotic spent medium. (**A**) Growth curve of *F. nucleatum* cultured in 0.5% BHIH (control) or in spent medium of probiotics (SM_IP_, dilution factors 1:9 to 1:1). (**B**) Growth curve of probiotic consortium cultured in BHI or in spent medium of *F. nucleatum* (SM_Fnp_). No significant inhibition was observed. (**C**–**H**) Inhibition of *F. nucleatum* growth by increasing concentrations of probiotic spent medium (dilution factors 1:9 to 1:1) measured at different time points. IP: intestinal probiotics; Fnp: *F. nucleatum*; SM: spent medium. (*n* = 3 biological replicates, * *p* < 0.05, ** *p* < 0.01, *** *p* < 0.001, **** *p* < 0.001, one-way ANOVA with Dunnett’s correction.)

The growth curve results show that probiotics and *F. nucleatum* entered the stationary phase at the 12th and 16th hours of culture, respectively (Figure 3A,B). The spent medium from probiotics significantly reduced the bacterial concentration of *F. nucleatum* in the stationary phase, and the inhibitory effect was dose-dependent, but did not affect the time taken to enter the stationary phase (Figure 3A,C–H). In contrast, the spent medium of *F. nucleatum* had no significant effect on the growth of probiotics (Figure 3B).

**Figure 4:**
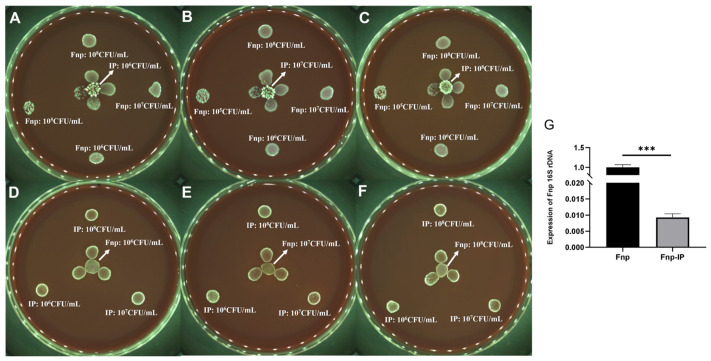
Direct growth inhibition of *F. nucleatum* by probiotics assessed via plate confrontation and qRT-PCR. (**A**–**F**) Representative images of BHI blood agar plates. For panels (**A**–**C**), the central spot is probiotics (10^6^–10^8^ CFU/mL), and the peripheral spot (40 mm away) is *F. nucleatum* (10^5^–10^8^ CFU/mL). For panels (**D**–**F**), the central spot is *F. nucleatum*, and the peripheral spot is probiotics. Negative controls (the same strain as the peripheral spot but placed alone at the edge of the plate) are shown in each panel. Increasing probiotic concentrations caused a clear zone of growth inhibition (**A**–**C**), whereas varying *F. nucleatum* concentrations did not inhibit probiotic growth (**D**–**F**). (**G**) The relative physiological activity and abundance of *F. nucleatum* were measured by means of 16S rRNA expression (qRT-PCR) after co-culture with probiotics. (*n* = 3 biological replicates, *** *p* < 0.001, *t*-test). Fnp-IP: *F. nucleatum* co-cultured with probiotics.

The plate confrontation experiment further confirmed that probiotics had a concentration-dependent inhibitory effect on the growth of *F. nucleatum* (Figure 4A–C), while *F. nucleatum* had no significant inhibition on probiotics (Figure 4D–F). qRT-PCR detection of *F. nucleatum* 16S rRNA gene expression in the co-culture system showed that its expression level in the Fnp-IP group was significantly lower than in the monoculture group (Figure 4G), suggesting that although *F. nucleatum* can integrate into the probiotic community and carry out metabolic activities, its bacterial count is significantly reduced, and its physiological activity is significantly inhibited.

### 3.3. Probiotic Metabolites Significantly Regulate the Gene Transcription Levels of F. nucleatum

**Figure 5:**
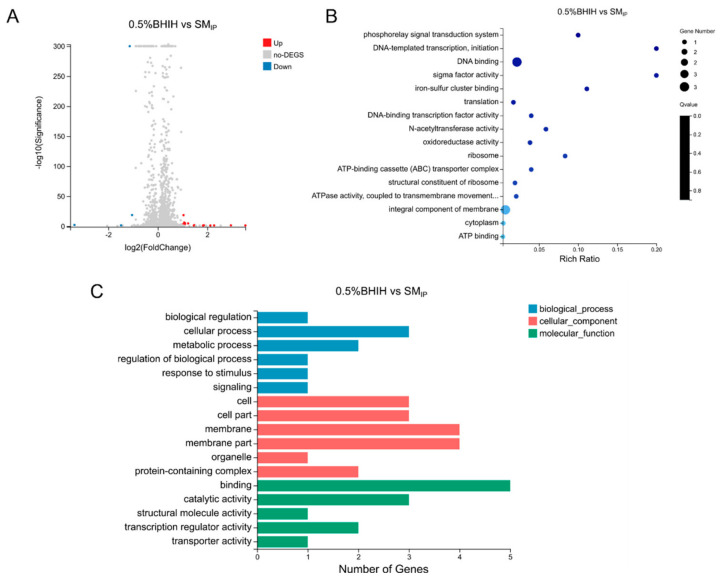
Transcriptomic response of *F. nucleatum* to probiotic spent medium. (**A**) Volcano plot of differentially expressed genes (DEGs) in *F. nucleatum* after exposure to probiotic spent medium (SM_IP_) compared to control (BHI). Red: upregulated genes; blue: downregulated. Six genes were upregulated, and two were downregulated. (**B**) GO enrichment analysis (biological process) of DEGs. (**C**) KEGG pathway classification. SM_IP_: probiotic spent medium. (*n* = 3 biological replicates, Log_2_FC > 1, FDR < 0.001.).

After treatment with probiotic supernatant, a total of eight genes in *F. nucleatum* showed significant expression changes, with six upregulated (including ABC transporter system ATP-binding protein, protoporphyrinogen III oxidase, GNAT family protein, LytTR family protein, and two genes of unknown function) and two downregulated (50S ribosomal protein L34 and 1 gene of unknown function) (Figure 5A). GO functional enrichment analysis showed that differentially expressed genes were mainly enriched in phosphorelay signal transduction system and DNA-templated transcription and initiation-related gene sets (Figure 5B,C). KEGG pathway classification indicated that these genes were mainly concentrated in the translation pathway.

### 3.4. Antibiotics Weaken the Inhibitory Effect of Probiotics on F. nucleatum and Affect Transcriptional Regulation

**Figure 6:**
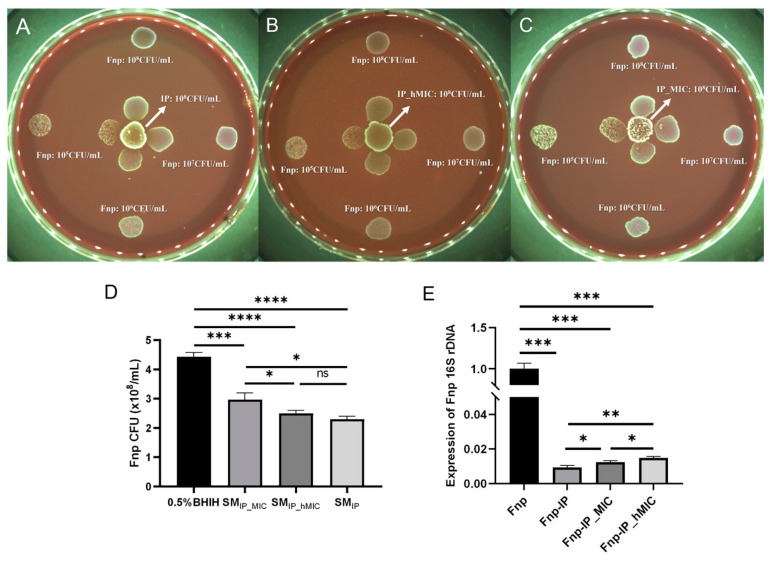
Antibiotic (ABX) treatment reduces the antagonistic activity of probiotics against *F. nucleatum*. (**A**–**C**) Plate confrontation assay. Central spot: probiotics untreated (**A**), pretreated with hMIC (**B**) or MIC (**C**) ABXs. Peripheral spot (40 mm away) and edge negative control: *F. nucleatum*. ABX pretreatment reduced the inhibition zone. (**D**) Spent medium assay. *F. nucleatum* CFU/mL after 24 h exposure to supernatants from untreated probiotics (SM_IP_) or probiotics pretreated with hMIC (SM_hMIC_) or MIC (SM_MIC_) ABXs. MIC ABX pretreatment reduced inhibitory activity (higher CFU vs. SM_IP_). (**E**) qRT-PCR of *F. nucleatum* 16S rRNA after co-culture with probiotics, untreated or pretreated with hMIC or MIC ABXs. Both ABX-treated groups showed higher 16S rRNA levels vs. the untreated co-culture, indicating increased physiological activity and relative abundance. (*n* = 3 biological replicates, * *p* < 0.05, ** *p* < 0.01, *** *p* < 0.001, **** *p* < 0.0001, one-way ANOVA with Tukey’s HSD.)

We found that the MIC of ABX against probiotics was 0.78 μg/mL and the hMIC was 0.39 μg/mL, while the MIC against *F. nucleatum* was below 0.195 μg/mL. Both plate confrontation experiments and spent medium experiments indicated that, after ABX treatment, the inhibitory effect of probiotics on *F. nucleatum* growth was weakened (Figure 6A,B). The qRT-PCR results further showed that, after ABX treatment, 16S rRNA gene expression of *F. nucleatum* was upregulated in the co-culture system (Figure 6C), indicating that antibiotics weakened the antagonistic effect of probiotics.

**Figure 7:**
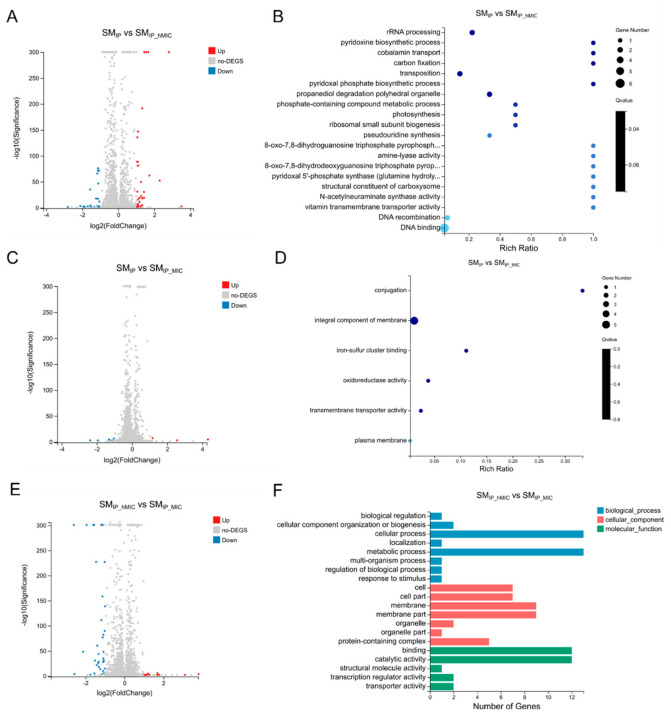
*F. nucleatum* transcriptome after exposure to probiotic supernatant from ABX-pretreated cultures. (**A**) Volcano plot of DEGs induced by SM_IP_ vs. SM_IP_hMIC_; 25 genes were upregulated, 11 were downregulated. (**B**) GO enrichment analysis of DEGs from (**A**). (**C**) Volcano plot of DEGs induced by SM_IP_ vs. SM_IP_MIC_; 5 were upregulated, 3 were downregulated. (**D**) GO enrichment of DEGs from (**C**). (**E**) Volcano plot of DEGs comparing SM _IP_MIC_ vs. SM_hMIC_; 2 were upregulated, 31 were downregulated. (**F**) GO functional classification of DEGs from (**E**). SM_IP_: untreated probiotic supernatant; SM__hMIC_ and SM_IP_MIC_: supernatants from probiotics pretreated with half-MIC or MIC of ABXs (*n* = 3 biological replicates, Log_2_FC > 1, FDR < 0.001).

Transcriptomic analysis revealed that the probiotic supernatant treated with ABX at hMIC (SM_IP-hMIC_) induced more gene expression changes in *F. nucleatum*; in particular, compared to the SM_IP_ group, nucleic acid metabolism, nucleotide metabolism, and propanediol degradation-related genes were upregulated 2.0–2.4-fold, acetylation-related genes were upregulated 2.6-fold, the pyridoxine synthesis gene *pdxS* was upregulated 6.8-fold, and transmembrane transport-related genes were downregulated 2.0–3.0-fold (Figure 7A). GO enrichment analysis suggested that DEGs were involved in multiple processes such as propanediol degradation polyhedral organelle, rRNA processing, and pyridoxine biosynthetic process (Figure 7B and Figure S2). KEGG analysis showed that these genes were significantly enriched in the sulfur metabolism pathway (Figure S3).

Compared to the SM_IP_ group, the probiotic supernatant treated with ABXs at MIC (SM_IP-MIC_) induced the upregulation of five genes and the downregulation of three genes in *F. nucleatum* (Figure 7C). GO enrichment analysis suggested that DEGs were involved in the integral component of membrane and iron–sulfur cluster binding (Figure 7D and Figure S4).

Compared to the SM_IP-hMIC_ group, SM_IP-MIC_ upregulated 2 genes and downregulated 31 genes in *F. nucleatum* (Figure 7E). GO functional classification analysis suggested that DEGs were involved in multiple processes, such as biological process, cellular process, and metabolic process (Figure 7F). KEGG functional classification suggested that these genes were involved in pathways such as cellular community–prokaryotes, membrane transport, metabolism, energy metabolism, and the metabolism of cofactors and vitamins (Figure S5).

### 3.5. ABX Alters the Community Composition of Probiotics in the Co-Culture System, with Significant Changes in the Abundance of L. plantarum and L. paracasei

**Figure 8:**
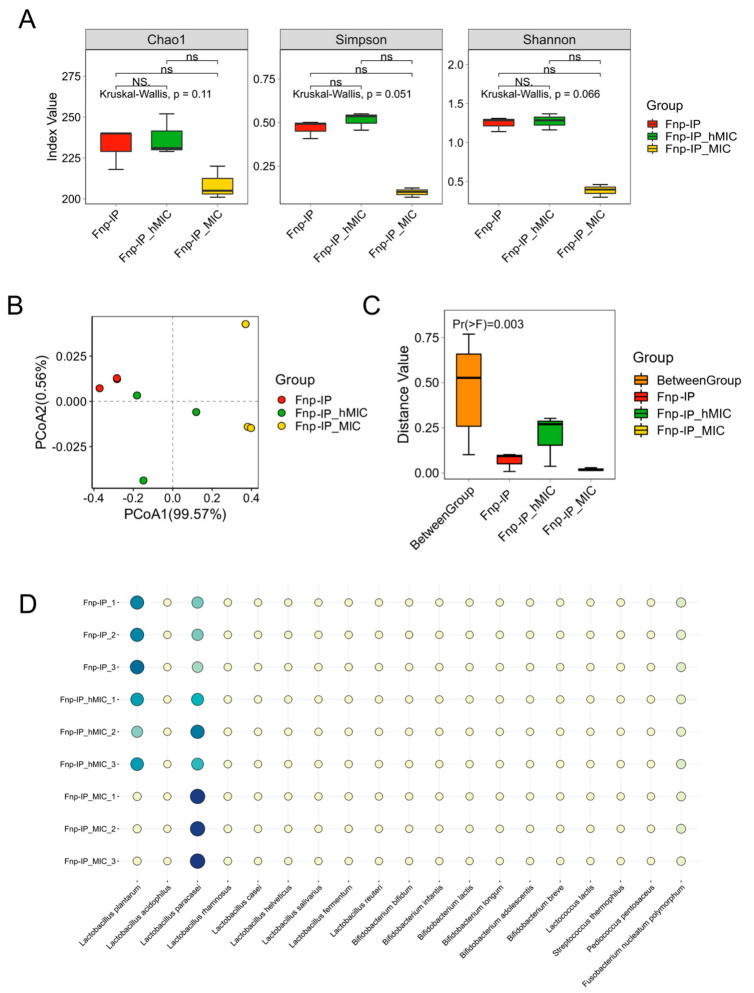
The effect of ABX pretreatment on the community composition of the probiotic consortium during co-culture with *F. nucleatum*. (**A**) The alpha diversity of Fnp-IP (co-culture without ABX), Fnp-IP_hMIC (half-MIC ABX pretreatment), and Fnp-IP_MIC (MIC ABX pretreatment). No significant difference among groups. (**B**) PCoA of beta diversity based on Bray–Curtis distance. Each dot represents one biological replicate (*n* = 3 per group). (**C**) PERMANOVA (999 permutations) revealed significant separation among the three groups (*p* = 0.003, R^2^ = 0.92). (**D**) Species abundance bubble plot. The relative abundance of *L. plantarum* (decreased) and *L. paracasei* (increased) changed significantly after ABX treatment. Bubble size represents the mean relative abundance.

Alpha diversity analysis showed no significant changes in species richness and evenness in the Fnp-IP_hMIC and Fnp-IP_MIC groups compared to the Fnp-IP group (Figure 8A). Beta diversity was visualized by PCoA based on the Bray–Curtis distance. PERMANOVA (999 permutations) confirmed a significant difference in overall community composition among the three groups (Figure 8B,C; *p* = 0.003, R^2^ = 0.92), indicating that ABX treatment significantly altered the community structure of the probiotic consortium.

The species abundance bubble plot shows that the species with the most significant changes after ABX treatment were *L. plantarum* and *L. paracasei*: the abundance of *L. plantarum* decreased, while that of *L. paracasei* increased (Figure 8D). MIC determination further confirmed that *L. plantarum* was more sensitive to ABX (MIC = 0.49 μg/mL), while *L. paracasei* was relatively resistant (MIC = 1.95 μg/mL).

### 3.6. L. plantarum and L. paracasei Cells and Their Metabolites Both Inhibit F. nucleatum Growth

**Figure 9:**
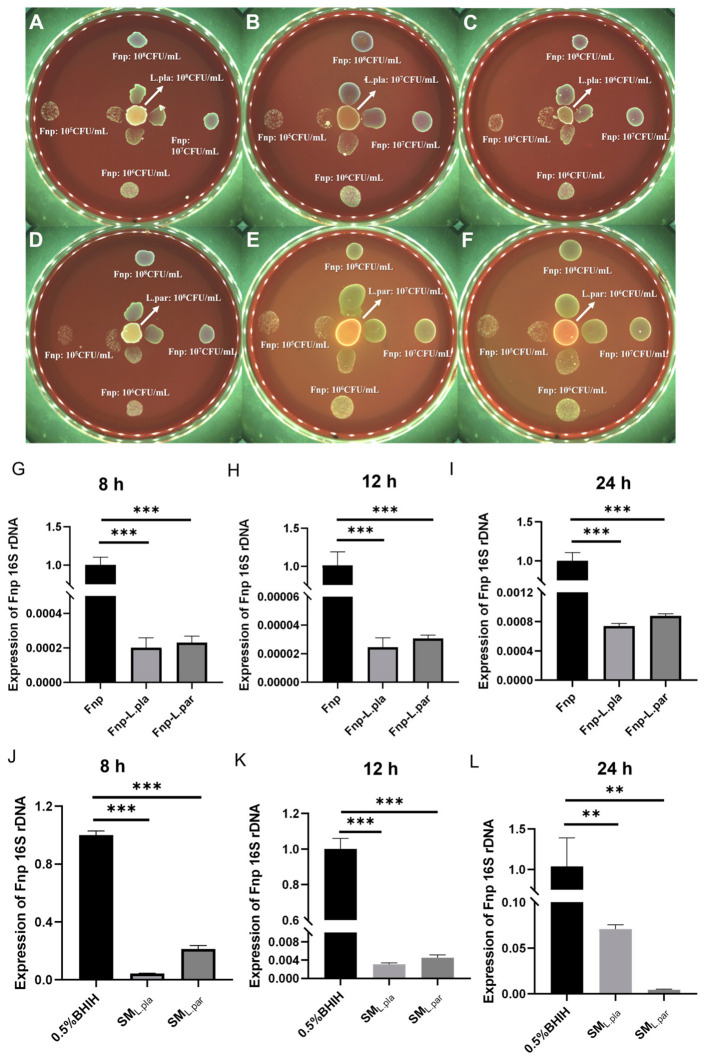
Two representative probiotic strains, *L. plantarum* and *L. paracasei*, inhibit *F. nucleatum* growth and activity. (**A**–**F**) Plate confrontation assay. Central spot: *L. plantarum* (**A**–**C**) or *L. paracasei* (**D**–**F**) (10^6^–10^8^ CFU/mL); peripheral spot (40 mm away) and edge negative control: *F. nucleatum* (10^7^ CFU/mL). Dose-dependent inhibition zones were observed. (**G**–**I**) qRT-PCR analysis of *F. nucleatum* 16S rRNA expression after co-culture with *L. plantarum* or *L. paracasei*, reflecting changes in both physiological activity and relative abundance. (*** *p* < 0.001, *t*-test). (**J**–**L**) Effect of CFS from *L. plantarum* or *L. paracasei* on *F. nucleatum* 16S rRNA expression. CFS significantly reduced *F. nucleatum* activity and load. (** *p* < 0.01, *** *p* < 0.001, *t*-test). L. pla: *L. plantarum*; L. par: *L. paracasei*; CFS: cell-free supernatant. *n* = 3 biological replicates.

The growth curves showed that *L. plantarum* and *L. paracasei* both entered the stationary phase at the 21st hour of culture (Figure S6). Plate confrontation experiments indicated that both exerted concentration-dependent inhibitory effects on *F. nucleatum* (Figure 9A–F). The qRT-PCR results showed that, in separate co-cultures with these two lactobacilli, 16S rRNA gene expression of *F. nucleatum* was significantly lower than that in the monoculture group (Figure 9G–I). In addition, their CFSs also significantly inhibited the physiological activity of *F. nucleatum* (Figure 9J–L).

**Figure 10:**
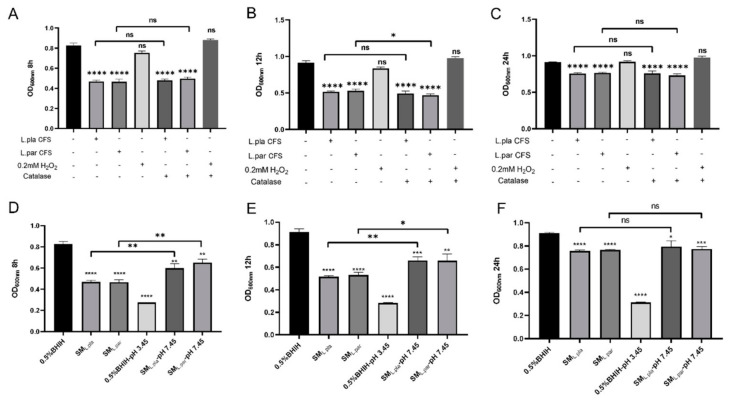
The effect of hydrogen peroxide and organic acids on the inhibitory activity of *L. plantarum* and *L. paracasei* CFS. (**A**–**C**) The growth of *F. nucleatum* in the CFS of *L. plantarum* or *L. paracasei* before and after hydrogen peroxide depletion (catalase treatment). No significant difference in growth. (**D**–**F**) The growth of *F. nucleatum* in the CFS before and after pH adjustment to 7.45 (neutralized). The inhibitory effect was partially lost at early time points (8 h, 12 h), but at 24 h the final cell density remained significantly lower than that of the control. Control: 0.5% BHIH medium with or without pH adjustment to 3.45. (*n* = 3 biological replicates, * *p* < 0.05, ** *p* < 0.01, *** *p* < 0.001, **** *p* < 0.0001, one-way ANOVA with Dunnett’s correction.)

To explore their antibacterial substances, we tested the effects of hydrogen peroxide and organic acids in the supernatant. After hydrogen peroxide was depleted, the antibacterial activity of the supernatant did not change significantly (Figure 10A–C), indicating that hydrogen peroxide was not the main antibacterial component. However, after adjusting the supernatant pH from 3.45 to 7.45, its inhibitory effect was significantly weakened in the early culture stages (8 h, 12 h); at 24 h, even after pH neutralization, the final bacterial concentration of *F. nucleatum* was still significantly lower than that of the control group (Figure 10D–F). This indicates that organic acids in the supernatant may inhibit *F. nucleatum* proliferation in the early growth phase and delay its entry into the stationary phase or reduce the bacterial concentration in the stationary phase.

### 3.7. Lysine Restores the Abundance of ABX-Treated Probiotics and Interferes with Coaggregation Between Probiotics and F. nucleatum

**Figure 11:**
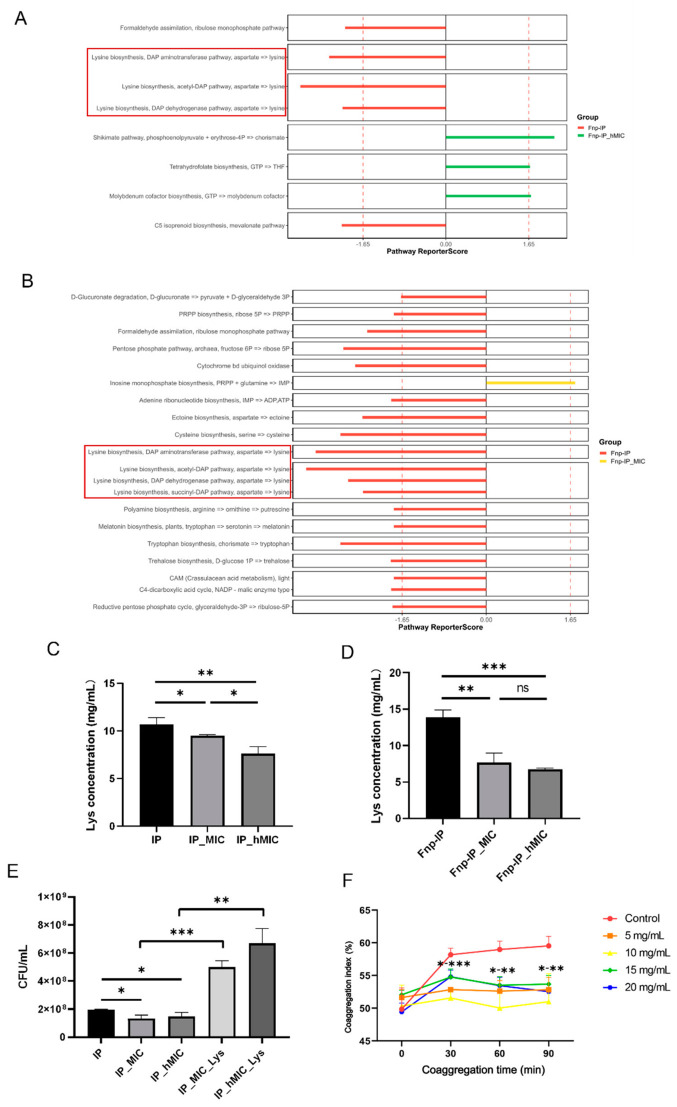
Lysine restores ABX-treated probiotics and reduces coaggregation with *F. nucleatum*. (**A**,**B**) KEGG pathway enrichment comparing Fnp-IP (red) vs. Fnp-IP_hMIC (green) (**A**) and Fnp-IP (red) vs. Fnp-IP_MIC (yellow) (**B**). The lysine biosynthesis pathway (red bar) was significantly enriched in Fnp-IP but lost after ABX treatment. (**C**,**D**) The lysine concentration in probiotic CFS (**C**) and in co-culture supernatants (**D**). ABX treatment significantly reduced lysine levels (*n* = 3, * *p* < 0.05, ** *p* < 0.01, *** *p* < 0.001, one-way ANOVA with Tukey’s HSD). (**E**) Total CFU counts of ABX-treated probiotics before and after lysine supplementation (11 mg/mL). Lysine restored probiotic abundance. (*n* = 3, * *p* < 0.05, ** *p* < 0.01, *** *p* < 0.001, *t*-test). The horizontal lines with asterisks (e.g., *-** or *-***) indicate significant differences between the indicated concentration groups. (**F**) The coaggregation index (CI) between probiotics and *F. nucleatum* at increasing lysine concentrations (0–20 mg/mL). Lysine dose-dependently reduced coaggregation (*n* = 3, *** *p* < 0.001, one-way ANOVA with Dunnett’s correction). Fnp-IP: co-culture without ABX; Fnp-IP_hMIC: half-MIC ABX pretreatment; Fnp-IP_MIC: MIC ABX pretreatment.

Metagenomic analysis showed that the lysine biosynthesis pathway was significantly enriched in the co-culture of probiotics and *F. nucleatum*, while the enrichment level of this pathway decreased after ABX treatment (Figure 11A,B). Experiments confirmed that the lysine concentration in the supernatant of ABX-treated probiotics was significantly reduced (Figure 11C,D), and the probiotic quantity also decreased; after lysine supplementation, the probiotic quantity was restored (Figure 11E).

Further measurements of the coaggregation index found that as lysine concentration increased, the coaggregation ability of *F. nucleatum* with probiotics significantly decreased (Figure 11F). Based on these findings, it is plausible that lysine alleviates the inhibitory effect of ABXs on probiotics and promotes colonization resistance, potentially through interference with interbacterial co-aggregation; however, further studies are required to confirm this mechanism.

## 4. Discussion

This study explores the interactions between *F. nucleatum* and intestinal probiotics and their potential significance in the occurrence and development of CRC. Through in vitro co-culture systems, we observed the impact of *F. nucleatum* on the composition and function of the probiotics and further investigated the inhibitory effects of these probiotics and their metabolites on the growth of *F. nucleatum* and the associated mechanisms. In addition, we examined the effects of antibiotic intervention on probiotic functions and the interactions between *F. nucleatum* and probiotics.

We found that, when *F. nucleatum* is co-cultured with probiotics, although it does not significantly inhibit the overall growth of the probiotics, it can alter their metabolic pathways. Mechanistic analysis through metagenomic sequencing revealed that, after co-culture of *F. nucleatum* with probiotics, pathways such as isoleucine, leucine biosynthesis, and histidine degradation in the microbial community were significantly downregulated. Beta diversity analysis (PCoA based on Bray–Curtis distance) did not detect a statistically significant shift in the overall community composition under our experimental conditions. This may be due to the limited sample size, which reduces statistical power to detect moderate changes, or to the fact that only a subset of species changed in abundance, and the changes in different species may have occurred in opposite directions, offsetting each other in the overall distance calculation. Nevertheless, previous studies have reported that, in mouse models colonized by *F. nucleatum*, the intestinal microbial composition was altered and related pathways such as amino acid metabolism and vitamin synthesis were downregulated without complete stagnation of microbial growth [42]. Supplementation with probiotics such as *L. plantarum* can restore homeostasis through negative regulation and promote the generation of beneficial metabolites [42]. These results suggest that *F. nucleatum* may primarily alter the probiotic community by inducing metabolic reprogramming rather than direct toxic killing.

In the metagenomic sequencing experiment, KEGG pathway enrichment analysis revealed that riboflavin synthesis and fatty acid synthesis were significantly enriched in the *F. nucleatum*–probiotic co-culture system. The literature indicates that probiotics can produce vitamin B2 through the riboflavin synthesis pathway, which can act as a signaling molecule involved in bacterial quorum sensing and host–bacteria interactions, improving the intestinal redox state and promoting anaerobic probiotic growth, thereby indirectly inhibiting the colonization of pathogens such as *F. nucleatum* [43,44]. In addition, short-chain fatty acids (such as butyrate) produced by probiotics through the fatty acid synthesis pathway can directly inhibit the growth of *F. nucleatum*, restore intestinal butyrate balance, enhance barrier function, and overcome chemotherapy resistance [45].

This study further found that the probiotic supernatant can significantly inhibit the growth of *F. nucleatum* and regulate the transcription levels of multiple genes. Among them, ABC transporter system ATP-binding protein, protoporphyrinogen III oxidase, and LytTR family protein gene expression were upregulated, while 50S ribosomal protein L34 gene expression was downregulated. Reports have indicated that, under acid–base stress, ABC transporter expression in *F. nucleatum* can be upregulated 2–3-fold to maintain ion and nutrient uptake and osmotic pressure balance [46]. Protoporphyrinogen III oxidase is a key enzyme in anaerobic heme synthesis, and its upregulation helps to cope with iron/oxidative stress, enhancing heme acquisition to support respiratory chain function [47]; the LytTR family protein also participates in regulating iron acquisition [48]. These changes suggest that the probiotic supernatant may force *F. nucleatum* to activate the anaerobic heme synthesis pathway in order to maintain redox homeostasis through mechanisms such as iron chelation. The downregulation of ribosomal protein L34 expression may reduce ribosome assembly, conserve energy, and regulate nucleocytoplasmic translocation [49], indicating that under the pressure of probiotic metabolites, *F. nucleatum* may slow protein synthesis and shift to a defensive state.

Antibiotic treatment may lead to gut microbiota dysbiosis and induce resistance gene production, thereby increasing the risk of CRC. Therefore, this study further explored the impacts of antibiotic treatment on probiotic inhibition against *F. nucleatum*. For this purpose, a broad-spectrum antibiotic “cocktail” combination, known to reduce intestinal microbial diversity and promote CRC development, was selected [50]. In clinical antibiotic therapy, blood concentrations often fluctuate, with prolonged periods at sub-MIC or near-MIC levels. At full MIC, probiotics may be partially killed, preventing effective supernatant production or observation of subsequent interactions. In contrast, hMIC allows for probiotic survival and the secretion of adaptive metabolites. Therefore, this study employed both MIC and hMIC concentrations to investigate the mechanisms of “residual resistance” and to simulate probiotic functional decline under gut microbiota dysbiosis after antibiotic treatment [51]. We found that, after 24 h of ABX treatment (MIC or hMIC), the direct and indirect inhibitory effects of probiotics on *F. nucleatum* were significantly weakened, indicating that antibiotics may disrupt the niche resistance of probiotics, creating an advantage for *F. nucleatum* growth.

Antibiotics also affected the transcriptional regulation of probiotic supernatant on *F. nucleatum*. In this study, we found that compared to the SM_IP_ group, the SM_IP-hMIC_ group led to significant upregulation of nucleotide metabolism, propanediol degradation, acetylation-related genes, and the pyridoxine synthesis gene *pdxS* in *F. nucleatum*. Studies have indicated that, under stress conditions such as hypoxia, nucleotide metabolism-related gene expression in *F. nucleatum* can be upregulated about 2-fold, helping to enhance its persistence in adverse environments [52]. In inflammatory intestinal environments, *F. nucleatum* can integrate symbiotic bacterial metabolism by upregulating propanediol/glycolysis degradation pathways (2–2.5-fold), competing for resources and generating energy [53]. In the σ^E stress response, acetyltransferase genes are upregulated 2–3-fold, modulating virulence and resistance through protein modification to cope with envelope or nutrient stress [54,55]. At the same time, in global stress responses, σ^E-mediated *pdxS*-like vitamin synthesis gene expression can be significantly upregulated about 6-fold. These transcriptional changes reflect the adaptive response of *F. nucleatum* to the probiotic supernatant after ABX treatment, involving σ^E-mediated global stress responses to enhance nutrient acquisition, detoxification, and protein modification capabilities, while downregulating non-essential transport processes to maintain survival. This adaptive mode may further strengthen the colonization and persistence of *F. nucleatum* in the intestinal or tumor microenvironment under antibiotic-induced dysbiosis.

Compared to the SM_IP-hMIC_ group, the SM_IP-MIC_ group showed significant downregulation of GNAT family protein-related and MarR family protein-related genes in *F. nucleatum*. These two types of genes are both related to environmental stress responses, which suggests that as the antibiotic concentration increases, the stress pressure caused by probiotic metabolites on *F. nucleatum* may further decrease, leading to downregulation of the expression of corresponding stress genes in *F. nucleatum*.

Within the probiotic community, the species with the most significant changes after ABX treatment were *L. plantarum* and *L. paracasei*, with the former’s abundance decreasing and the latter’s increasing. Although our metagenomic analysis does not resolve individual strains, this does not compromise the subsequent single-strain validation experiments, which confirmed the differential ABX sensitivity of *L. plantarum* and *L. paracasei*. This may stem from *L. plantarum*’s greater sensitivity to certain components in ABXs (such as β-lactams), while *L. paracasei* can gain a relative advantage in competition through resistance mechanisms such as membrane changes or metabolic adaptations [56,57]. These two lactobacilli, as important intestinal probiotics, can antagonize pathogens through multiple mechanisms, including niche competition, bacteriocin secretion, short-chain fatty acids, organic acids, and hydrogen peroxide [58].

We also found that the main active substances in the CFS of *L. plantarum* and *L. paracasei* inhibiting *F. nucleatum* are organic acids. The CFS of both probiotics can inhibit the proliferation of *F. nucleatum* in the early growth phase, prolong its lag phase, and reduce the bacterial concentration upon entering the stationary phase. The literature reports that organic acids in lactobacillus CFS can extend the lag phase of *F. nucleatum* by 2–6 h and inhibit its division rate, leading to a 20–60% decrease in stationary phase bacterial concentration [59,60]. The associated mechanism may involve organic acids diffusing into bacterial cells, depleting ATP, and inhibiting key enzyme activities such as glycolysis, rather than direct bactericidal action, thereby helping probiotics to gain an advantage in intestinal ecological competition [59,61].

*F. nucleatum*, an anaerobic bacterium capable of migrating from the oral cavity to the gastrointestinal tract, resists gastric acid (HCl, pH ≈ 1.5) primarily through membrane lipid remodeling, including the synthesis of erucic acid to enhance membrane stability and maintain cellular integrity [62]. Although resistant to this strong acid, it remains susceptible to probiotic-derived weak organic acids (e.g., lactic acid and SCFAs such as butyric, propionic, and acetic acids), which inhibit its growth via multiple pathways [63,64]. Unlike HCl, which fully dissociates into impermeable H^+^ and Cl^−^ ions, exerting mainly extracellular stress, weak organic acids (pKa ≈ 3.8–4.8) exist predominantly in their undissociated, lipophilic form (HA) at acidic extracellular pH. This neutral HA passively diffuses across the lipid bilayer without transporters or ion pumps [65]. Once inside the cell, HA dissociates, causing intracellular acidification, disruption of proton motive force, ATP depletion, and inhibition of glycolysis—explaining their superior inhibitory effect on F. nucleatum colonization compared to strong acids [65].

This study found that the lysine synthesis pathway was significantly enriched when probiotics were co-cultured with *F. nucleatum*, while the enrichment of this pathway decreased after ABX treatment. Studies have indicated that antibiotics often interfere with amino acid synthesis and uptake in probiotics, leading to lysine depletion (20–50% decrease), thereby inhibiting protein synthesis and growth; lysine supplementation can alleviate this effect by enhancing proton motive force or buffering pH, promoting probiotic abundance recovery [66,67,68]. In addition to the lysine pathway, other metabolic pathways (e.g., those related to nucleotide metabolism, stress responses, and vitamin synthesis) also showed altered enrichment after ABX treatment (Figure 11A,B), which warrant further investigation in future studies.

Lysine plays an important role in biological metabolism, growth, and development, and is an essential amino acid for maintaining overall health and normal physiological functions of the body [69]. Lysine acetylation is currently one of the most widespread forms of post-translational protein modification known in bacteria, which is involved in various regulatory processes such as bacterial transcription, translation, metabolism, and stress responses [70,71,72]. Cohort studies have shown that blood lysine levels are negatively associated with the risk of colorectal cancer [73]. Intestinal probiotics can convert lysine into butyrate while releasing ammonium ions, serving as an energy source for colon cells and helping to prevent inflammation and cancer development [74,75,76]. Meanwhile, lysine may interfere with its mediated interbacterial coaggregation by competitively binding to the adhesin RadD of *F. nucleatum* [77], thereby inhibiting *F. nucleatum*’s cross-niche colonization and integration into the gut microbiota. It has been reported that lysine can inhibit the expression and oligomerization of the hemolytic toxin Hla in *Staphylococcus aureus* (*S. aureus*), thereby reducing its virulence expression and colonization in food models (such as milk). In mouse and Caco-2 cell models, lysine alleviates the intestinal damage caused by *S. aureu*s, demonstrating that it can prevent the colonization of pathogenic bacteria in food and the intestine by reducing bacterial attachment and virulence [78].

In periodontal disease patients with *F. nucleatum* overgrowth [79], the bacterium may translocate via the oral–gut axis to colonize the intestine, thereby promoting CRC initiation and progression [3,4]. Our findings demonstrate that lysine significantly inhibits *F. nucleatum*’s coaggregation with probiotics, likely by competitively binding to the adhesin RadD on the bacterial surface. This disruption impairs *F. nucleatum*’s ability to integrate into the gut microbial community, facilitating its clearance from the intestine rather than persistent colonization. Therefore, lysine supplementation or lysine-enriched probiotic strategies hold promising practical value as a non-antibiotic intervention to reduce *F. nucleatum*-mediated CRC risk in high-risk populations, such as those with chronic periodontitis.

Probiotics show potential for CRC prevention by inhibiting *F. nucleatum* colonization in the gut microbiota. Strains such as *Bifidobacterium* and *Lactobacillus* reduce the abundance of *F. nucleatum* through pH modulation, antimicrobial activity, and barrier formation, thereby attenuating CRC-associated inflammation and tumorigenesis [80,81]. This resistance to pathogenic colonization enhances microbial diversity and immune responses, offering a novel adjunct strategy for CRC prophylaxis [82]. Future clinical trials should validate these mechanisms in high-risk populations to optimize probiotic interventions.

We acknowledge that this study is in vitro and used a single *F. nucleatum* subspecies (subsp. *polymorphum*) and one antibiotic cocktail at fixed concentrations. The complexity of the 33-strain probiotic consortium means that our findings represent one plausible scenario among many possible ones. Different *F. nucleatum* subspecies (e.g., *animalis*, *vincentii*, *watanabei*), other antibiotic types, concentrations, or exposure times could yield different outcomes. Moreover, the metagenomic sample size was limited; thus, larger cohorts are needed to strengthen the conclusions. Additionally, the probiotic supernatant likely contains additional antimicrobial molecules (e.g., bacteriocins, reuterin) beyond those examined here; methods such as high-performance liquid chromatography (HPLC) would be required for full identification. Future in vivo studies using clinically relevant isolates and defined consortia are required to validate and extend our conclusions.

## 5. Conclusions

This study shows that probiotics and their metabolites inhibit the growth of *F. nucleatum* and modulate its stress- and metabolism-related genes. Antibiotic treatment weakens this inhibition and changes the stress response of *F. nucleatum*, which may enhance its adaptability in dysbiotic environments. Organic acids from *L. plantarum* and *L. paracasei* are key inhibitory effectors. Exogenous lysine restores antibiotic-reduced probiotic abundance and reduces coaggregation with *F. nucleatum*, suggesting a possible role in colonization resistance.

Together, these results suggest that probiotics might help to counteract *F. nucleatum* colonization via organic acids and metabolic interference, potentially contributing to gut homeostasis. However, antibiotic use could compromise this protective effect, which might favor the persistence of *F. nucleatum* and could thus be relevant to CRC risk. Further studies are needed to confirm these hypotheses in more complex systems.

## Data Availability

The original contributions presented in this study are included in the article/Supplementary Material. Further inquiries can be directed to the corresponding authors. The raw data of the metagenomic sequencing were deposited in the Sequence Read Archive (SRA) under Bioproject accession numbers PRJNA1415463 and PRJNA1415878. The RNA sequencing data supporting the findings of this study were deposited in the NCBI SRA under accession number PRJNA1415531.

## Supplementary Materials

The following supporting information can be downloaded at: https://www.mdpi.com/article/10.3390/microorganisms14050965/s1. Figure S1: The library preparation strategy; Figure S2: Bubble plots of GO enrichment analysis (SM_IP_ vs. SM_IP_hMIC_); Figure S3: Bubble plots of KEGG pathway enrichment analysis (SM_IP_ vs. SM_IP_hMIC_); Figure S4: GO functional classification (SM_IP_ vs. SM_IP_MIC_); Figure S5: KEGG functional classification analysis (SM_IP_hMIC_ vs. SM_IP_MIC_); Figure S6: Growth curves of *L. plantarum* and *L. paracasei* in BHI. Table S1: Gene-specific primer sequences.
