# Supplementary material for: Probiotics Exert Colonization Resistance Against F. nucleatum subsp. polymorphum: Disruption by Antibiotics and Underlying Molecular Mechanisms
Source: Microorganisms. 2026 Apr 24;14(5):965. doi: 10.3390/microorganisms14050965 (PMC13210348; doi:10.3390/microorganisms14050965)
Supplement: Supplementary file 1 [file microorganisms-14-00965-s001.zip › Table S1.pdf]

Gene ID	Primer sequences (5’-3’)
Fnp-16S rRNAgene	F：CTTAGGAATGAGACAGAGATG
	R：TGATGGTAACATACGAAAGG
LDJ86_RS00895	F：AGTAGCACCTTATACCACGAAAA
	R：TGCTCCAGTTGGTGAGAAAA
LDJ86_RS02290	F：TCAGGTGGAGAGTTACAAAAAGC
	R：TTTGCCTCTTGTGCCAATGC
LDJ86_RS02295	F：ATTGGAGTTGGAGCAGGTGG
	R：TCAAACTGCATTAAGCCTGAAATC
LDJ86_RS03440	F：TGGTACAAGCACCCAAACCA
	R：AGATGGAGTGAAAGACCCTCT
LDJ86_RS03570	F：TGTAGCTCATGTTTAGTGTCCA
	R：GTGCTCTGGAAGGAACTAAGGT
LDJ86_RS03645	F：GTTGCACCTTAGTCATGGCA
	R：TCAATAGCAAGGGGTTTGGCT
LDJ86_RS04275	F：CACATGAAGCCTTTGCACCA
	R：ATGTGCTGCTTCTGGTGTTG
LDJ86_RS04980	F：TGGTGCTTCATGCCCTCCTA
	R：TGGATAGATATGCCTCTGCACT
LDJ86_RS07025	F： AGTTGCCATTGTTCCTCCGAA
	R：GGTGCTGATGTTGTTGCAGT
LDJ86_RS07520	F： TGTTCCGTCAATCTCAAAAGTAGT
	R： AATGGGGAGAAAGAAAACTTGC
LDJ86_RS09285	F： AAGTCCTGTTATCGCCCCTG
	R： TTTCCCTTTGGGAGCAGCAA
LDJ86_RS09530	F：TCATGGTTTTAGAGCTAGAATGTCA
	R：GCTCTTCCTCTAACTCTTCTTCT
LDJ86_RS09565	F：TGGCAGGAGTATATTTTATGGGTG
	R： GTGCCATAGGACAATCAGCA

LDJ86_RS10665	F：AGGGCATATTCCTATTTTGGCA
	R：GGAGTTACATAAGTTACACCAGGA
LDJ86_RS11145	F：ACTCGTCTTCCCGTAATGCT
	R：AAAGGGGAAGCTGGAACTGG
LDJ86_RS11840	F： AGCAGGGCTTGATGCAATTCT
	R：TGCCCTTATTGGTAATTCACGG
