# Supplementary figures and images for: Probiotics Exert Colonization Resistance Against F. nucleatum subsp. polymorphum: Disruption by Antibiotics and Underlying Molecular Mechanisms
Source: Microorganisms. 2026 Apr 24;14(5):965. doi: 10.3390/microorganisms14050965 (PMC13210348; doi:10.3390/microorganisms14050965)

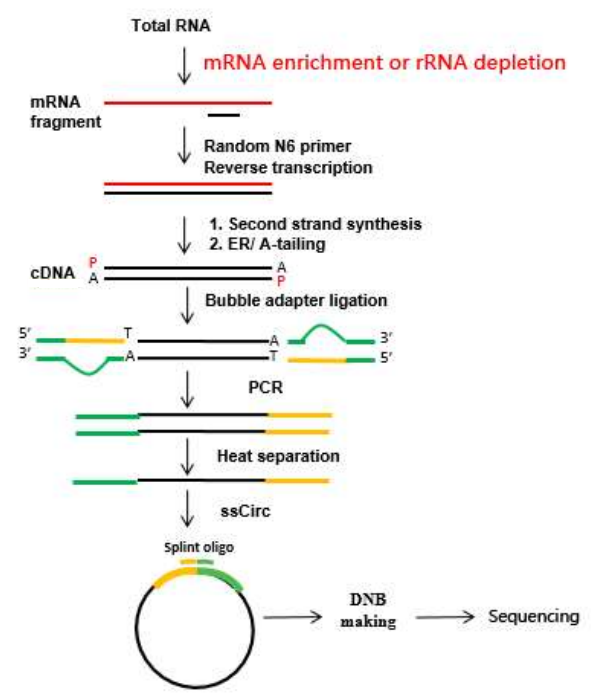

Supplement: Supplementary file 1 [file microorganisms-14-00965-s001.zip › Figure S1.png]

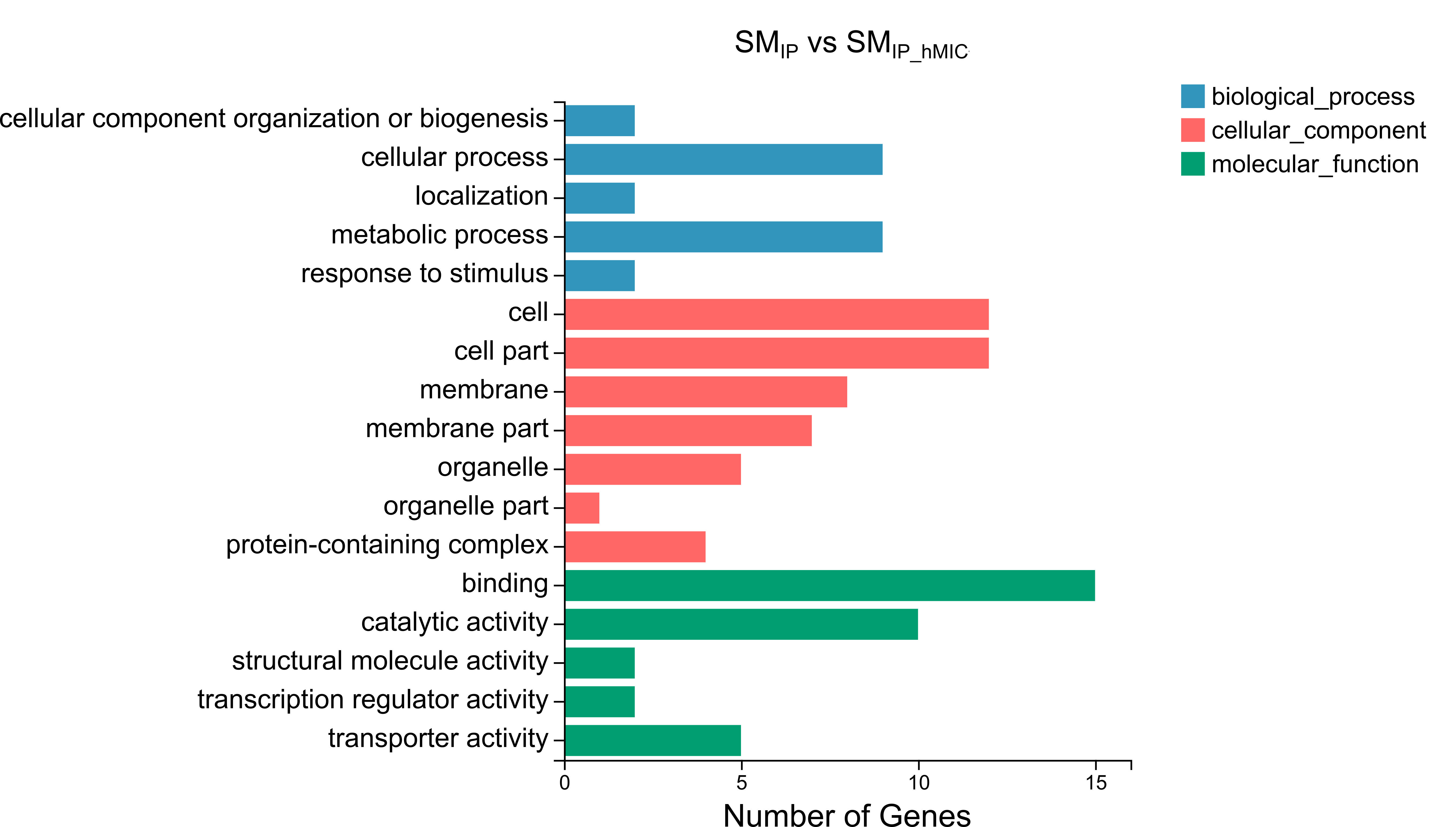

Supplement: Supplementary file 1 [file microorganisms-14-00965-s001.zip › Figure S2.tif]

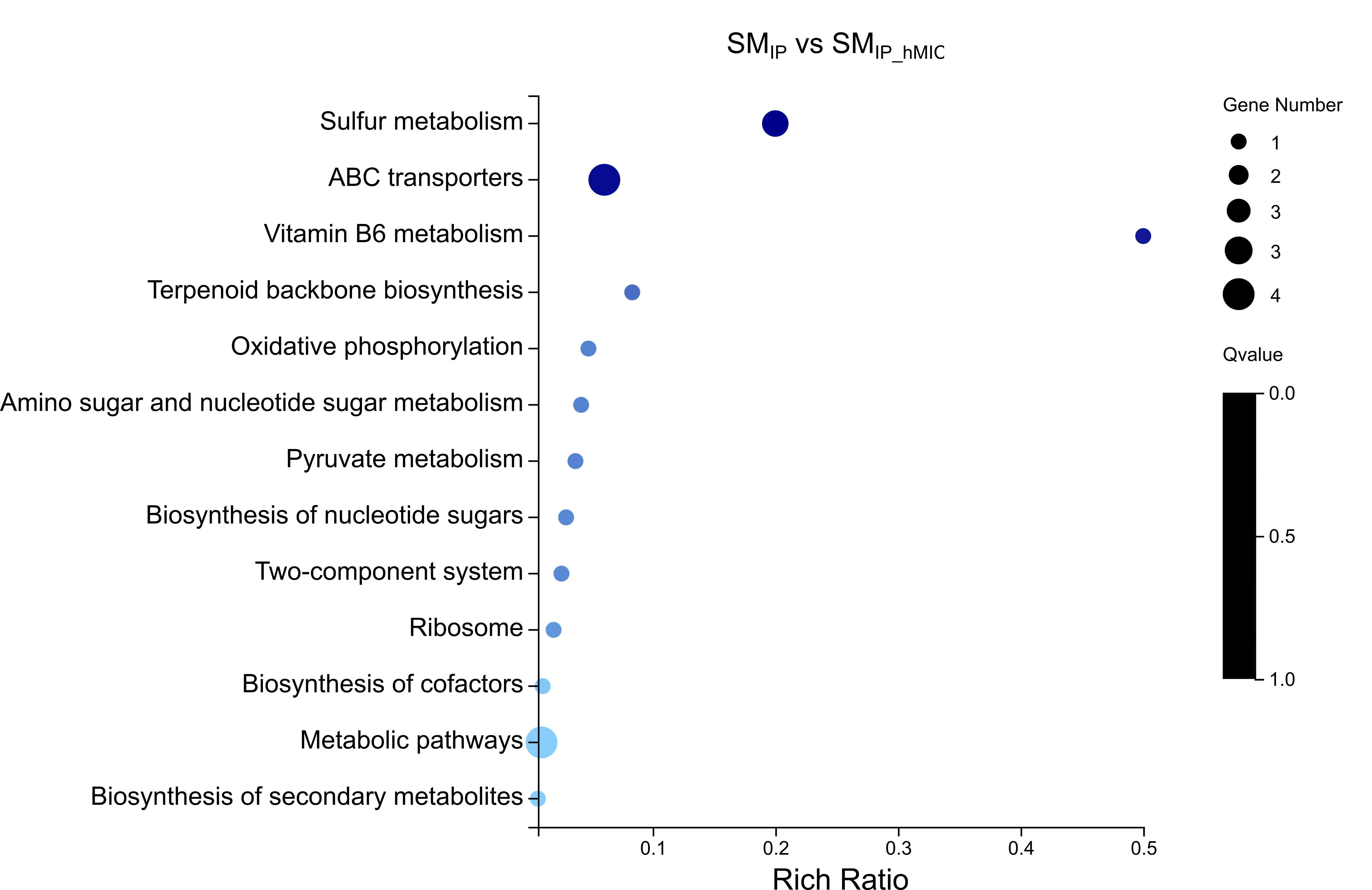

Supplement: Supplementary file 1 [file microorganisms-14-00965-s001.zip › Figure S3.tif]

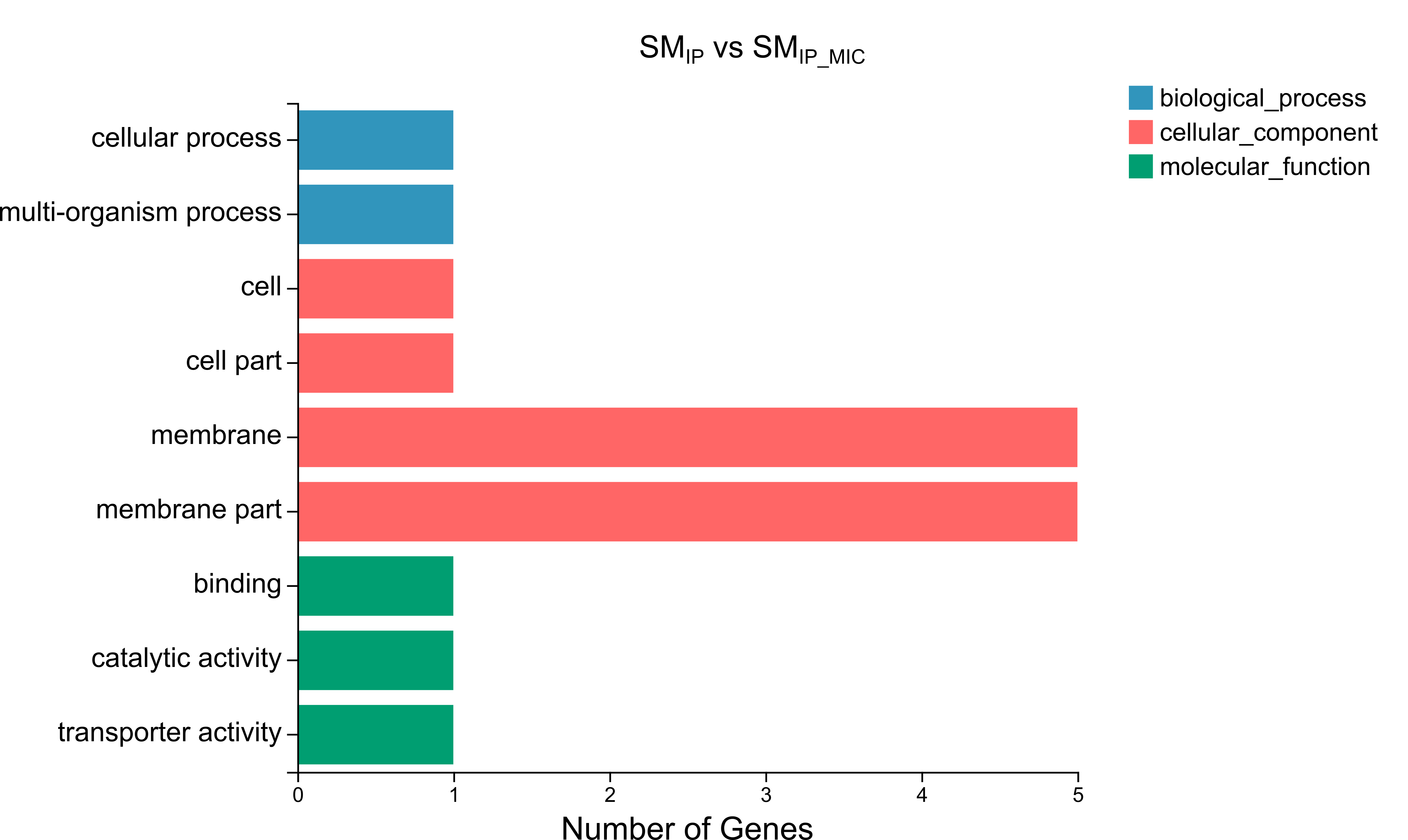

Supplement: Supplementary file 1 [file microorganisms-14-00965-s001.zip › Figure S4.tif]

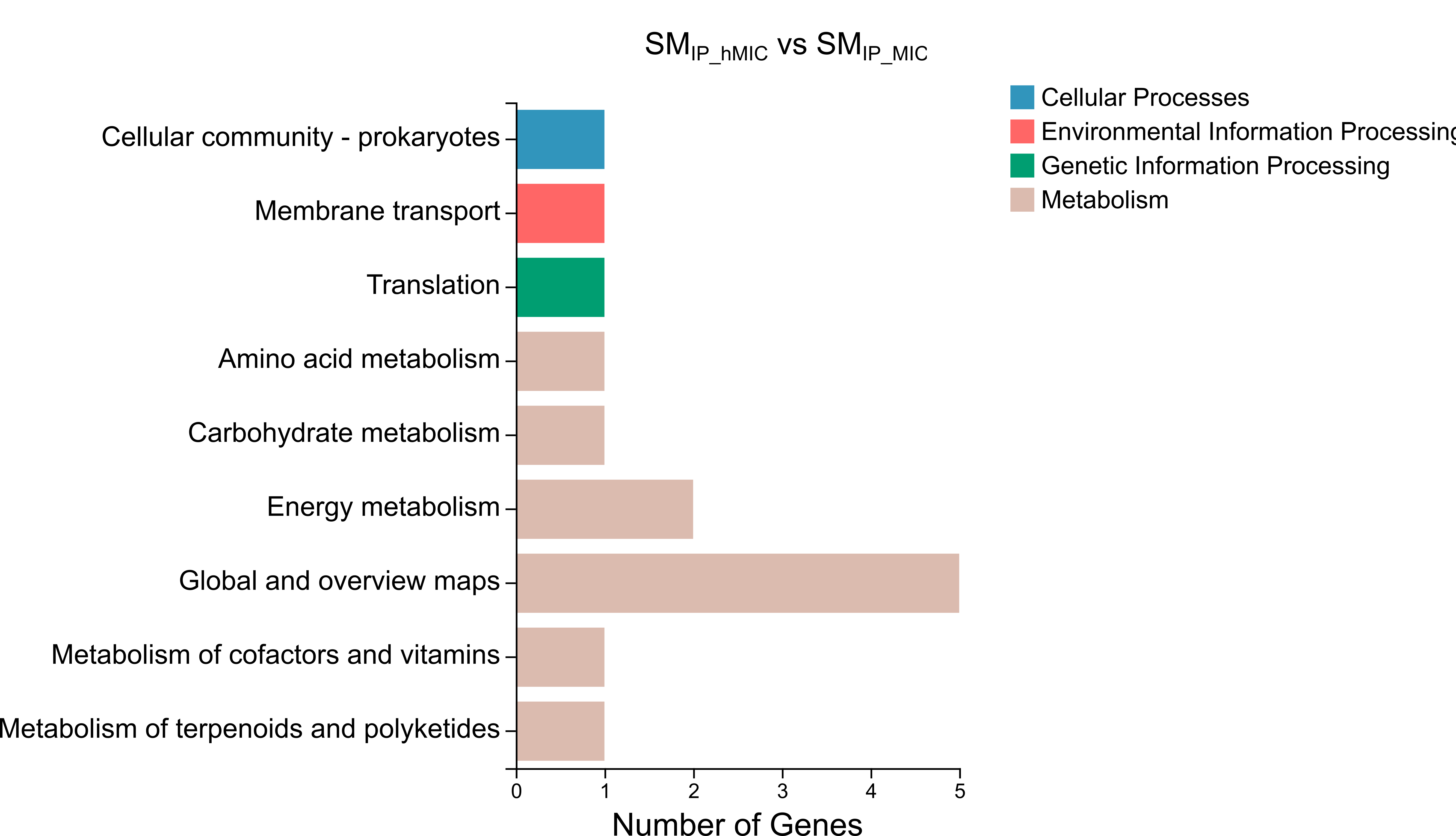

Supplement: Supplementary file 1 [file microorganisms-14-00965-s001.zip › Figure S5.tif]

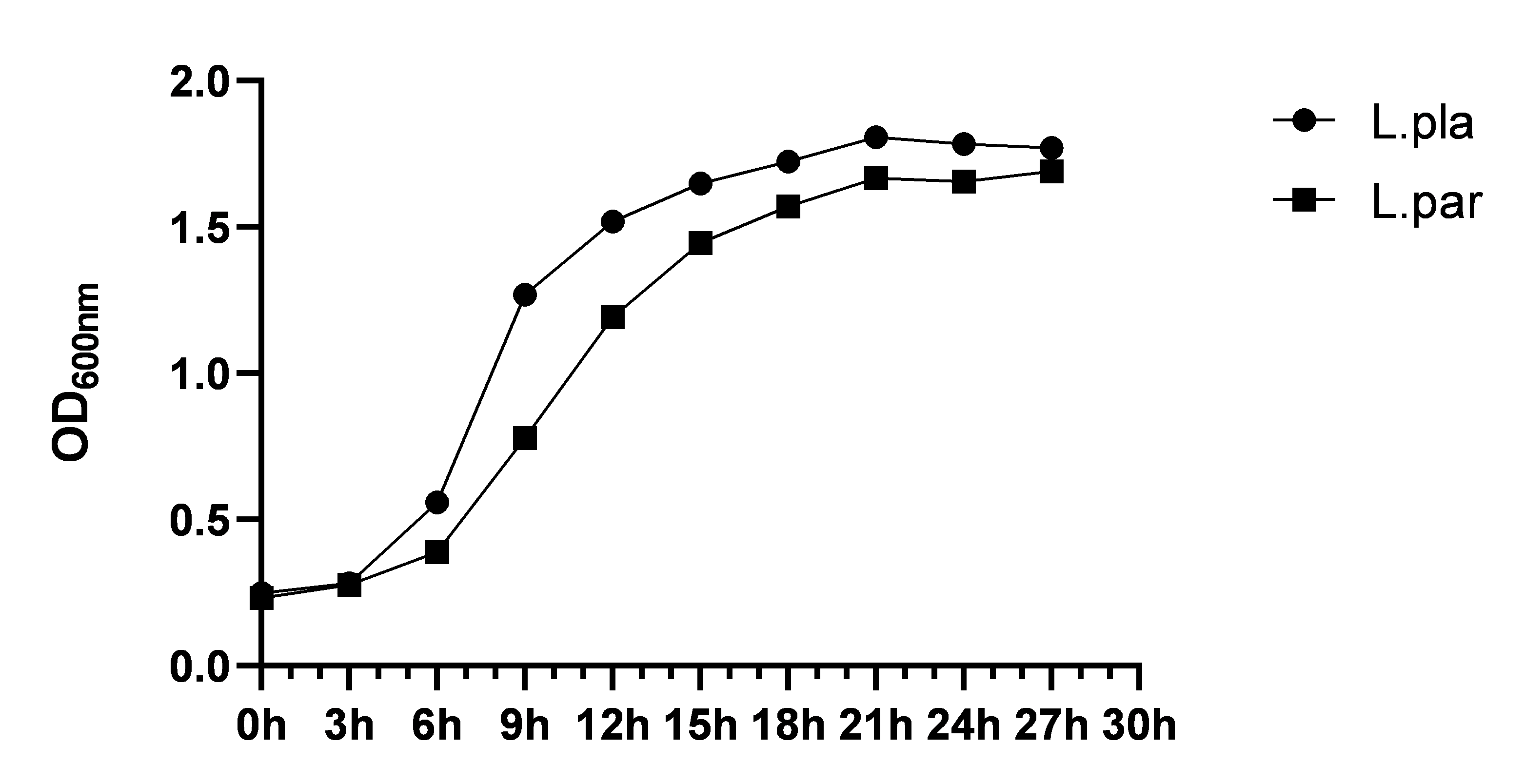

Supplement: Supplementary file 1 [file microorganisms-14-00965-s001.zip › Figure S6.tif]
